# Left Atrial Appendage Occlusion versus Novel Oral Anticoagulation for Stroke Prevention in Atrial Fibrillation—One-Year Survival

**DOI:** 10.3390/jcm12206693

**Published:** 2023-10-23

**Authors:** Shmuel Tiosano, Ariel Banai, Wesam Mulla, Ido Goldenberg, Gabriella Bayshtok, Uri Amit, Nir Shlomo, Eyal Nof, Raphael Rosso, Michael Glikson, Victor Guetta, Israel Barbash, Roy Beinart

**Affiliations:** 1 Leviev Heart Center, Sheba Medical Center, Ramat Gan 52621, Israelwesam.mulla@sheba.health.gov.il (W.M.);; 2 Faculty of Medicine, Tel Aviv University, Ramat Aviv, Tel Aviv 6997801, Israel; arielbanai@gmail.com (A.B.);; 3 Department of Cardiology, Tel Aviv Sourasky Medical Center, Tel Aviv 64239, Israel; 4 Surgeon General Headquarters, Israel Defense Forces, Ramat Gan 5262000, Israel; 5 Arrow Program, Sheba Medical Center, Ramat Gan 5266202, Israel; 6 Jesselson Integrated Heart Center, Shaare Zedek Medical Center, Jerusalem 9103102, Israel; 7 Faculty of Medicine, Hebrew University of Jerusalem, Jerusalem 9574425, Israel

**Keywords:** left atrial appendage occluder, NOAC, survival, cohort, anticoagulation

## Abstract

Aim To compare the 1-year survival rate of patients with atrial fibrillation (AF) following left atrial appendage occluder (LAAO) implantation vs. treatment with novel oral anticoagulants (NOACs). Methods: We have conducted an indirect, retrospective comparison between LAAO and NOAC registries. The LAAO registry is a national prospective cohort of 419 AF patients who underwent percutaneous LAAO between January 2008 and October 2015. The NOACs registry is a multicenter prospective cohort of 3138 AF patients treated with NOACs between November 2015 and August 2018. Baseline patient characteristics were retrospectively collected from coded diagnoses of hospitalization and outpatient clinic notes. Follow-up data was sorted from coded diagnoses and the national civil registry. Subjects were matched according to propensity score. Baseline characteristics were compared using Chi-Square and student’s *t*-test. Survival analysis was performed using Kaplan-Meier survival curves, log-rank test, and multivariable Cox regression, adjusting for possible confounding variables. Results: This study included 114 subjects who underwent LAAO implantation and 342 subjects treated with NOACs. The mean age of participants was 77.9 ± 7.44 and 77.1 ± 11.2 years in the LAAO and NOAC groups, respectively (*p* = 0.4). The LAAO group had 70 (61%) men compared to 202 (59%) men in the NOAC group (*p* = 0.74). No significant differences were found in baseline comorbidities, renal function, or CHA_2_DS_2_-VASc score. One-year mortality was observed in 5 (4%) patients and 32 (9%) patients of the LAAO and NOAC groups, respectively. After adjusting for confounders, LAAO was significantly associated with a lower risk for 1-year mortality (HR 0.38, 95%CI 0.14–0.99). In patients with impaired renal function, this difference was even more prominent (HR 0.21 for creatinine clearance (CrCl) < 60 mL/min). Conclusions: In a pooled analysis of two registries, we found a significantly lower risk for 1-year mortality in patients with AF who were implanted with LAAO than those treated with NOACs. This finding was more prominent in patients with impaired renal function. Future prospective direct studies should further investigate the efficacy and adverse effects of both treatment strategies.

## 1. Introduction

Atrial fibrillation (AF), the most common sustained cardiac arrhythmia, affects 3% of the general population and one-fourth of the elderly [1]. It increases the risk of ischemic stroke [2]. Similarly, the prevalence of chronic kidney disease (CKD) increases with age [3], rising to 25% in patients older than 70 [4], and is associated with an increased risk of AF, independent of age [5,6]. Patients with both conditions have a higher risk of ischemic stroke or bleeding [7], making management challenging.

Until a decade ago, warfarin was the preferred drug for preventing ischemic stroke in AF patients [8,9], but it has a narrow therapeutic range, requires frequent monitoring, has numerous drug and food interactions, and poor patient compliance [10]. With the introduction of novel oral anticoagulants (NOACs) for the treatment of non-valvular AF (NVAF) [11,12,13], the use of warfarin has decreased, except for certain indications such as mechanical heart valve prosthesis or rheumatic mitral valve disease [14]. NOACs have the advantage of not needing monitoring and dose adjustments and fewer interactions with food and drugs.

The optimal anticoagulant for patients with CKD is still a matter of debate, with warfarin being recommended in guidelines [15] but real-world data showing reluctance to prescribe due to bleeding risk and fear of a fatal outcome [16]. NOACs have dose adjustments for CKD patients [17], but randomized trials in severe CKD are lacking. Left atrial appendage occlusion (LAAO) is an alternative invasive treatment with demonstrated benefits in randomized studies and registries [18,19]. The PLAATO trial evaluated the feasibility and safety of LAAO in patients unable to be treated with anticoagulation [20]. The PROTECT AF proved its non-inferiority at one year compared to warfarin [21]. In the follow-up publication, the LAAO showed superiority over warfarin, with reduced total mortality [22]. In the PREVAIL trial, procedural safety was significantly improved compared to PROTECT AF [23]. A Danish study had shown that LAAO might have similar stroke prevention efficacy but a lower risk of major bleeding and mortality [24].

Furthermore, the LAAOS III trial showed promising results in preventing stroke by surgically closing the left atrial appendage during cardiac surgery [25]. In the PRAGUE-17 randomized trial, LAAO was non-inferior to NOACs in preventing major AF-related cardiovascular, neurological and bleeding events, however, without a significant difference in survival [26]. The EHRA/EAPCI currently favor NOACs over LAAO in patients eligible for both treatments due to stronger evidence towards NOACs. Moreover, this position paper questions the PREVAIL study due to the higher rate ratio (1.33, 95% CI 0.78–2.13) of 18-month stroke seen as part of the first co-primary endpoint—a composite of stroke, systemic embolism, and cardiovascular/unexplained death. The recently published SCAI/HRS Expert Consensus Statement on Transcatheter Left Atrial Appendage Closure deems LAAO appropriate when patients are at high thromboembolic risk and not suited for long-term OAC [27]. This study evaluated the efficacy and safety of percutaneous LAAO compared to NOACs in patients with NVAF in an all-comers cohort. Specifically, it evaluated the subpopulation of patients with CKD.

## 2. Methods

### 2.1. Patients

This study compared patients who were treated with NOACs and those who were implanted with LAAO. The NOAC group is a pooled sample of 3 different separate multicenter prospective cohorts of consecutive patients treated with a NOAC (either Rivaroxaban *n* = 1023, Apixaban *n* = 1164, and Dabigatran *n* = 951) who entered the study between November 2015 and August 2018. A secure web-based questionnaire produced by the study coordination center at the Israeli Center for Cardiovascular Research was used for data collection. The LAAO database consists of 419 patients with AF who underwent percutaneous LAAO implantation between January 2008 and October 2015. Patients underwent propensity score matching with a 1:3 ratio. The final analysis included 342 patients from the NOAC group and 114 patients in the LAAO group. CrCl for this study was calculated based on the Cockroft-Gault formula.

Devices used for LAA occlusion were either Amplatzer Amulet (Abbott Cardiovascular, Plymouth, MN, USA) or Watchman (Boston Scientific, Marlborough, MA, USA). The decision on which type of device would be installed was based on the operating physicians’ preference and clinical characteristics. Usually, patients were discharged with a dual antiplatelet therapy consisting of Aspirin and Clopidogrel for six weeks, followed by a lifelong monotherapy with Aspirin. Most patients who underwent LAAO occlusion had contraindications for anticoagulant treatment.

### 2.2. Endpoints

The primary endpoint evaluated all-cause mortality at one year. Subgroup analysis of the primary endpoint for different baseline covariates, including renal function, was then executed. Secondary endpoints included ischemic stroke/transient ischemic attack rates and any bleeding event at 1-year follow-up.

### 2.3. Statistical Analysis

Statistical analysis was performed using R Version 3.6.3 (R Foundation for Statistical Computing, Vienna, Austria). Categorial variables are expressed as percentages and continuous variables are expressed as mean ± SD. Variables were compared using a t-test for continuous variables and a Chi-square for categorical variables. The Kaplan-Meier method was employed to calculate the probability of achieving the primary and secondary endpoints. The Cox regression model was used to calculate the hazard ratio for the primary endpoint while adjusting for potential confounders. *p* values < 0.05 were considered significant.

### 2.4. Propensity Score Matching

To ensure comparability between the LAAO and NOAC groups concerning potential confounding factors, we employed a logistic regression model to calculate the conditional propensity score. This encompassed baseline and clinical characteristics, including age, gender, hypertension, diabetes mellitus, IHD, and CHF, while the treatment group (LAAO or NOAC) served as the dependent variable. Subsequently, we conducted matching based on these conditional propensity scores and evaluated the balance using the standardized mean difference (SMD), with a threshold of SMD < 0.1 indicating a satisfactory balance.

## 3. Results

### 3.1. Study Population and Characteristics

Following propensity score matching, our cohort included 456 patients, 342 patients in the NOAC group and 114 in the LAAO group (Table 1). As for patients with CrCl ≤ 60 mL/min, our analysis included 279 patients, 217 patients in the NOAC group and 62 in the LAAO group (Table S1). All patients had paroxysmal, persistent, or permanent AF with an indication for anticoagulation according to the ESC guidelines. The mean age was 77.3 ± 10.4 years, and 59.6% were males. A sizable portion of patients had comorbidities, including hypertension (83%), diabetes (41%), ischemic heart disease (52%), congestive heart failure (37%), and a history of stroke or Transient Ischemic Attack (TIA) (39%). The mean CHA_2_DS_2_-VASc score was 4.02 ± 1.5, and the mean HAS-BLED score was 4.00 ± 1.3. Notably, 75% of the patients had a history of bleeding. The mean creatinine clearance was 54.4 ± 29.5 mL/min for the NOACs group and 58.3 ± 31.6 mL/min for the LAAO group (*p* = 0.24).

### 3.2. Primary Endpoint

**Figure 1:**
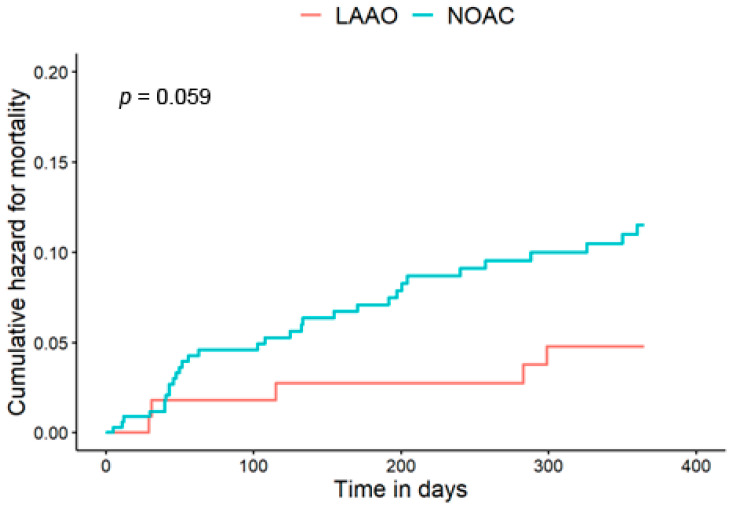
Kaplan-Meier curves comparing 1-year mortality of LAAO vs. NOACs. LAAO: Left Atrial Appendage Occlusion; NOACs: Novel Oral Anticoagulants.

**Figure 2:**
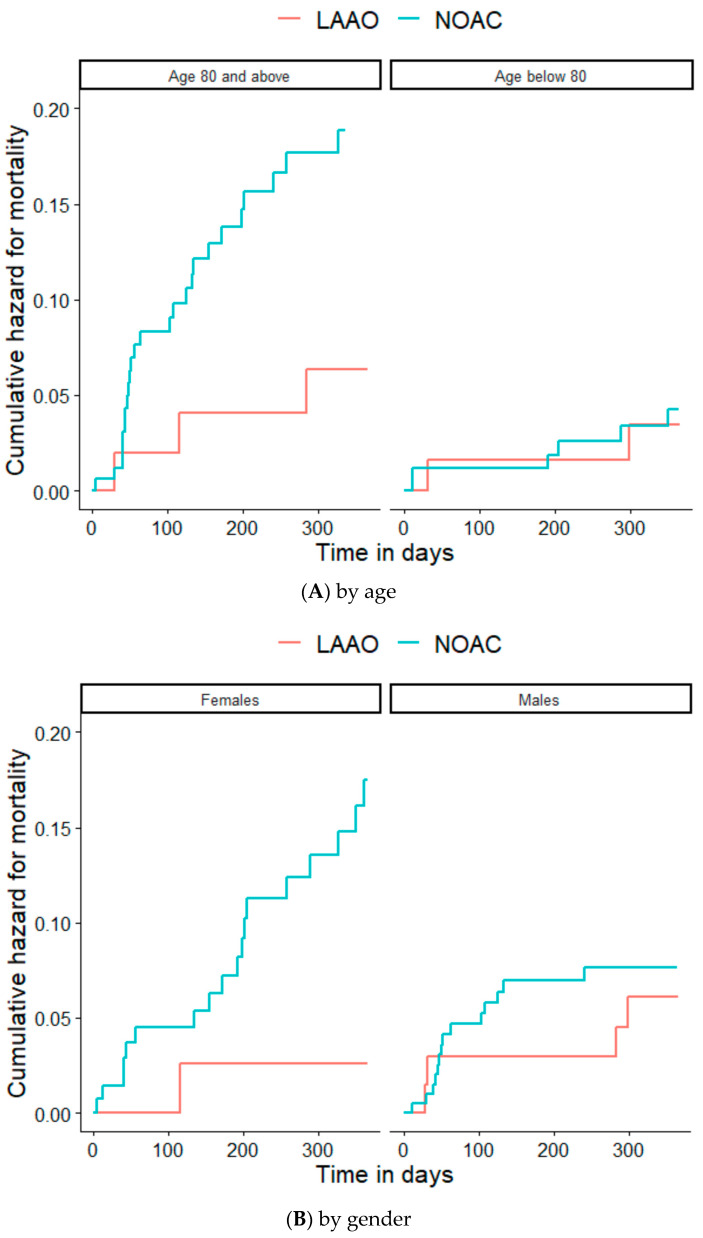

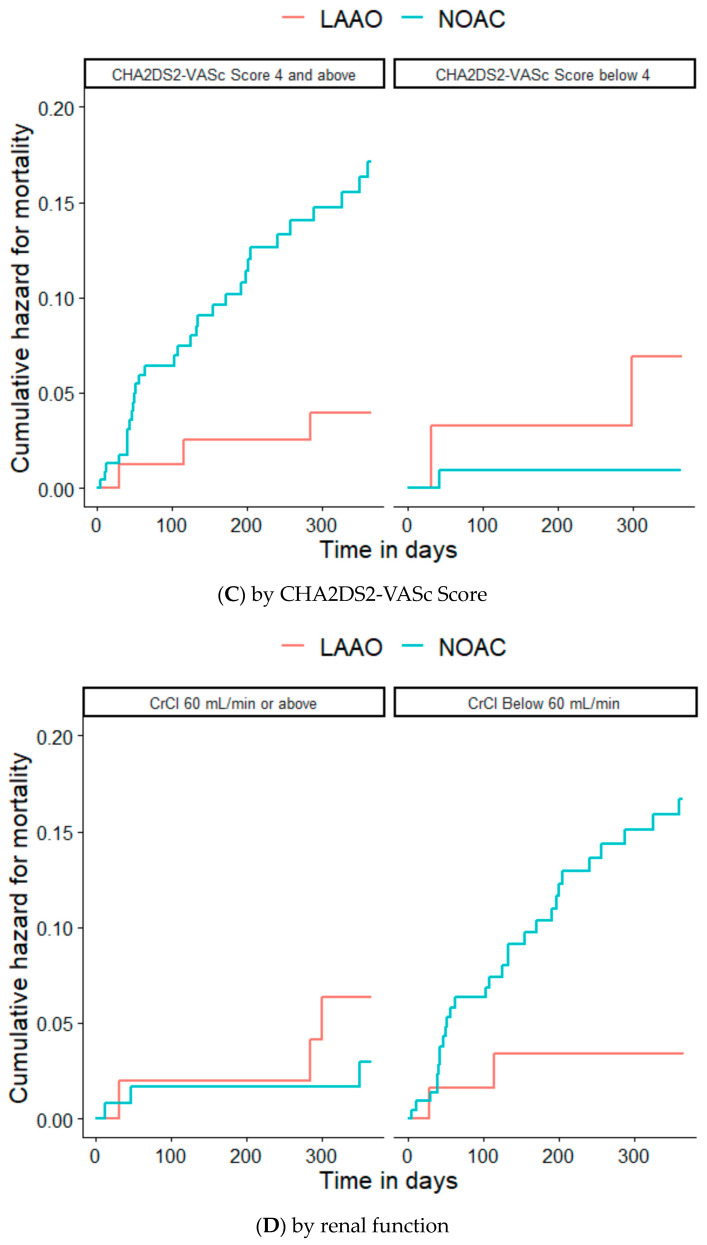
Kaplan-Meier curves comparing 1-year mortality of LAAO vs. NOACs by subgroups. LAAO: Left Atrial Appendage Occlusion; NOACs: Novel Oral Anticoagulant.

The primary endpoint, all-cause 1-year mortality, occurred in 37 patients—32 (9%) from the NOAC group and 5 (4%) from the LAAO (log-rank *p* = 0.059). Kaplan-Meier survival curves are presented in Figure 1. There was a significant interaction between reduced renal function and treatment group for crude mortality rates: For patients with CrCl ≤ 60 mL/min, all-cause 1-year mortality occurred in 29 (13%) vs. 2 (3%) in NOAC and LAAO group, respectively (*p* = 0.04), and for patients with CrCl > 60 mL/min 3 (2%) vs. 3 (6%) for NOAC and LAAO group, respectively (*p* = 0.36) (Table S2, Figure 2).

**Figure 3:**
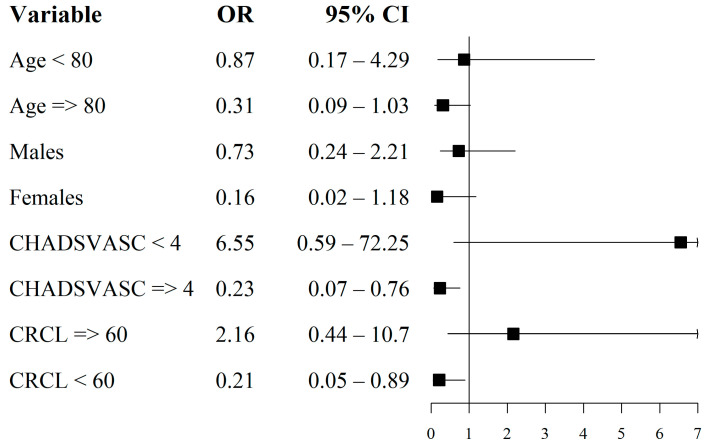
Subgroup analysis of factors associated with 1-year mortality for those who underwent LAAO implantation. LAAO: Left Atrial Appendage Occlusion; OR: Odds Ratio; CI: Confidence Interval; y: Years; CrCl: Creatinine Clearance.

Table 2 shows the multivariable Cox proportional hazard analysis results by group while adjusting for age, sex, hypertension, diabetes mellitus, ischemic heart disease, heart failure, prior bleeding, prior stroke or TIA, renal function, CHA_2_DS_2_-VASc, and HAS-BLED scores as well as prior aspirin treatment. LAAO implantation was significantly associated with reduced risk for 1-year mortality (HR 0.38, 95%CI 0.14–0.99) compared to treatment with NOACs. Interestingly, in the subgroup analysis (Figure 3), those who underwent LAAO implantation with CrCl of <60 mL/min, CHA_2_DS_2_-VASc score ≥ 4, and/or those who were on a reduced NOAC dose presented with a significantly lower adjusted risk for 1-year mortality (HR 0.21, 0.23 and 0.3, respectively).

### 3.3. Secondary Endpoints

At one year of follow-up, a total of 7 patients experienced stroke or TIA, 5 (1%) in the NOAC group and 2 (2%) in the LAAO group (*p* = 1.00). Likewise, major bleeding occurred in 15 patients, nine using NOACs and six in the LAAO group, without a significant between-group difference (3% vs. 5% respectively, *p* = 0.22)). Major bleeding was defined as a decrease in hemoglobin levels of 2 g/dL or more or bleeding that required a blood transfusion. Minor bleeding was defined as every bleeding which does not correspond to the definition of major bleeding. Prior bleeding was defined as any event of past major bleeding.

## 4. Discussion

Oral anticoagulation is recommended as the first-line therapy for preventing ischemic stroke in patients with AF and a CHA_2_DS_2_-VASc ≥ 1 in males and ≥2 in females [14]. LAAO initially emerged as an alternative therapeutic strategy for patients unable to tolerate anticoagulation or those who preferred to avoid long-term use. It proved its benefits over warfarin in several randomized controlled trials [21,22,23]. In the ESC guidelines, percutaneous LAAO is recommended only in patients with contraindications to anticoagulation [27,28].

In this study, which consisted of a non-direct comparison between LAAO and NOAC registries, we have demonstrated a statistically significant reduced risk for 1-year all-cause mortality in patients who underwent LAAO implantation compared to those treated with NOACs.

The results demonstrate that 1-year mortality is reduced in LAAO patients compared to NOACs, consistent with previous studies with propensity score-matched populations [24]. This finding also aligned with the PLAATO study (4.39% in the current study vs. 5.4% in PLAATO). The stroke rate in one year for the LAAO group presented in our study was comparable to the reported rate in PLAATO (1.75% vs. 1.8%, respectively). These results further support previous experience with LAAO regarding safety and efficacy in preventing stroke as reported based on the Amulet observational registry [18]. Another propensity-score matched study, which included patients with a previous ischemic stroke, compared 299 patients who underwent LAAO occlusion to 301 patients who received NOACs [29]. This analysis yielded a significantly lower (HR = 0.48) risk of the primary composite outcome of ischemic stroke, major bleeding, and all-cause mortality. The authors reported a surprisingly high rate of NOAC discontinuation of nearly 50% in 2 years. They stated that this may be one of the possible mechanisms that created differences between study arms. In a recently published real-world comparative analysis of Medicare claims data, LAAO implantation was associated with reduced risk for death, stroke, and long-term bleeding [30]. Our study correlates well with these results; however, we used a different study design—the matching ratio of 1:3 (LAAO:NOACs) vs. 1:1 in this study, and we also used the subdivision for renal failure and elderly age, whilst in the other study the gender effect was emphasized.

In this study, we performed an interaction analysis that revealed that our findings are more prominent among patients with renal failure, higher ischemic risk, and reduced NOAC dose.

### 4.1. Renal Failure

Renal failure is an important factor associated with increased risk for cardioembolic events and major bleeding. As such, it may play a role in the clinical decision to implant an LAAO or to medically treat eligible patients with NOACs. Lega and colleagues have demonstrated a linear relationship between the percentage of renal excretion of NOAC and the risk of major bleeding [31] while demonstrating the advantage of NOACs over warfarin. Thus, when tailoring patient-specific treatment, one should consider the inevitable course of deterioration in renal function that would eventually preclude the ability to use NOACs. Our study demonstrated a subgroup analysis with a reduced risk for 1-year all-cause mortality in patients taking reduced-dose NOACs. Under these circumstances, LAAO could be a reasonable alternative in patients with advanced kidney disease or patients with labile renal function.

There is an ongoing debate about whether treating ESRD patients with oral anticoagulants provides stroke protection over bleeding risk [32,33]. Accordingly, in the NOAC seminal studies, patients with ESRD were excluded. ESRD patients who opted to take oral anticoagulants were traditionally treated with warfarin. However, recent attempts were made to prescribe apixaban for ESRD patients, with modest evidence for the superiority of NOACs over warfarin in terms of major bleeding [34]. In our study, we had no patients with ESRD, and we cannot draw any specific conclusions. However, when considering the conflicting evidence for NOAC treatment in ESRD patients, in addition to our results in patients with CKD, LAAO could be a suitable treatment alternative in ESRD patients with NVAF.

### 4.2. Prior Major Bleeding

Patients with prior major bleeding were also underrepresented in the pivotal trials. The current study shows that these patients tolerated LAAO instead of NOACs (which are contraindicated in most cases) without elevated risk for rebleeding or stroke. Similarly, our study participants had higher CHA_2_DS_2_-VASc and HAS-BLED scores compared to the pivotal studies (4 in current study vs. 3.48, 2.1, and 2.1 in ROCKET-AF, RE-LY, and ARISTOTLE, respectively), [35] implying our study population is more similar to real-world data [36] and that our results may be generalized to other high-risk populations.

### 4.3. Low Dose NOACs

Patients with guideline-based indications for NOAC dose reduction are at increased risk for thrombotic and hemorrhagic complications despite being treated with anticoagulants [37]. A critical factor in determining the need for dose reduction is renal function, which also impacts the CHA_2_DS_2_-VASc score (indirectly by increasing risk for various cardiovascular diseases) and HAS-BLED score. The patient population with a significant advantage of using LAAO in the interaction analysis—e.g., those with an indication for reduced NOAC dose and those with increased bleeding and thrombotic risk—was the population with the most prominent cardiovascular disease burden. This phenomenon could be explained by the increased efficacy of LAAO over NOACs in stroke prevention (for example, when using a reduced NOAC dose) or by preventing unnecessary hemorrhagic phenomena among patients with labile or reduced renal function.

### 4.4. Limitations

Our study has several limitations. First, it is a retrospective study that utilized data from two different registries. The inclusion and exclusion criteria for the registries differ, resulting in diverse groups. We tried to mitigate this issue by applying propensity score matching. Second, as this study is based on registries, there is an inevitable loss of follow-up and inconsistency in event definition and validation. As such, we could not differentiate between major and minor bleeding. Thus, we decided to include all bleeding events regardless of severity, anatomical location, and the possible need for red blood cell transfusion. A third limitation of the study is the relatively short follow-up duration of one year. Another limitation is that we did not analyze the possible complications of LAAO implantation (such as cardiac perforation, pericardial effusion with tamponade, ischemic stroke, device embolization, and other vascular complications) [38]. In addition, we did not include information regarding the death cause of study participants. Lastly, both groups had a relatively low number of events, diminishing the power to detect other differences.

## 5. Conclusions

This pooled analysis of two registries demonstrated a lower risk for 1-year mortality among patients with AF who were implanted LAAO than those treated with NOACs. This result was more prominent in patients with reduced renal function. Future prospective randomized studies with larger sample sizes, ESRD and longer follow-up durations should further investigate the efficacy and adverse effects of both treatment strategies.

## Figures and Tables

**Table 1:** Baseline characteristics of study participants.

	LAAO *N* = 114	NOACs *N* = 342	*P*
Age	77.9 ± 7.44	77.1 ± 11.2	0.409
Sex: Male	70 (61.4%)	202 (59.1%)	0.741
Hypertension	98 (86.0%)	279 (81.6%)	0.353
Diabetes Mellitus	50 (43.9%)	137 (40.1%)	0.545
Ischemic Heart Disease	62 (54.4%)	173 (50.6%)	0.552
Congestive Heart Failure	44 (38.6%)	124 (36.3%)	0.737
Ejection fraction (%)	52.5 ± 7.27	52.9 ± 11.1	0.674
Prior bleeding	85 (74.6%)	256 (74.9%)	1.000
Prior stroke or TIA	49 (43.0%)	127 (37.1%)	0.317
CrCl	58.3 ± 31.6	54.4 ± 29.5	0.243
CrCl ≤ 60 mL/min	62 (54.4%)	217 (63.5%)	0.108
CHA_2_DS_2_-VASc	4.17 ± 1.29	3.96 ± 1.57	0.173
CHA_2_DS_2_-VASc ≥ 4	83 (72.8%)	230 (67.3%)	0.322
HAS-BLED	4.14 ± 1.04	3.95 ± 1.35	0.113
HAS-BLED ≥ 4	86 (75.4%)	236 (69.0%)	0.235
Prior Aspirin treatment	49 (43.0%)	126 (36.8%)	0.291

LAAO: Left Atrial Appendage Occlusion; NOACs: Novel Oral Anticoagulants; TIA: Transient Ischemic Attack; CrCl: Creatinine Clearance, mL/min.

**Table 2:** Multivariate analysis for factors associated with 1-year mortality.

	HR (95% CI)	*P*
Age above 80	3.23 (1.25, 8.39)	0.016
Sex: Female	1.48 (0.51, 4.33)	0.474
Group: LAAO	0.38 (0.14, 0.99)	0.048
Congestive Heart Failure	3.87 (1.30, 11.54)	0.015
Hypertension	0.39 (0.10, 1.43)	0.154
Diabetes Mellitus	1.63 (0.52, 5.15)	0.404
Ischemic Heart Disease	1.36 (0.67, 2.77)	0.401
Ejection fraction *	0.99 (0.96, 1.02)	0.655
Prior bleeding	4.39 (0.79, 24.32)	0.09
Stroke or TIA	1.30 (0.42, 4.00)	0.645
CrCl **	0.98 (0.96, 1.00)	0.123
CHA_2_DS_2_-VASc	1.08 (0.48, 2.42)	0.856
HAS-BLED	0.89 (0.46, 1.73)	0.741
Aspirin treatment	1.11 (0.48, 2.58)	0.807

LAAO: Left Atrial Appendage Occlusion; TIA: Transient Ischemic Attack; CrCl: Creatinine Clearance; * Per 1 percent increment; ** Per 1 mL/min increment.

## Data Availability

The datasets for this study cannot be made available due to the nature of personal information they contain.

## Supplementary Materials

The following supporting information can be downloaded at https://www.mdpi.com/article/10.3390/jcm12206693/s1. Table S1—Baseline characteristics of study participants with CrCl <= 60 mL/min, Table S2—The association between LAAO vs. NOACs and 1-year mortality – stratification by distinct groups.

## References

[1] Roger V.L., Go A.S., Lloyd-Jones D.M., Adams R.J., Berry J.D., Brown T.M., Carnethon M.R., Dai S., de Simon G., Wylie-Rosett J. (2011). Heart disease and stroke statistics—2011 update: A report from the American Heart Association. Circulation.

[2] Wolf P.A., Abbott R.D., Kannel W.B. (1991). Atrial fibrillation as an independent risk factor for stroke: The Framingham Study. Stroke.

[3] Coresh J., Selvin E., Stevens L.A., Manzi J., Kusek J.W., Eggers P., Van Lente F., Levey A.S. (2007). Prevalence of Chronic Kidney Disease in the United States. JAMA.

[4] Soliman E.Z., Prineas R.J., Go A.S., Xie D., Lash J.P., Rahman M., Ojo A., Teal V.L., Jensvold N.G., Robinson N.L. (2010). Chronic kidney disease and prevalent atrial fibrillation: The Chronic Renal Insufficiency Cohort (CRIC). Am. Heart J..

[5] Alonso A., Lopez F.L., Matsushita K., Loehr L.R., Agarwal S.K., Chen L.Y., Soliman E.Z., Astor B., Coresh J. (2011). Chronic kidney disease is associated with the incidence of atrial fibrillation: The Atherosclerosis Risk in Communities (ARIC) study. Circulation.

[6] Baber U., Howard V.J., Halperin J.L., Soliman E.Z., Zhang X., McClellan W., Muntner P. (2011). Association of chronic kidney disease with atrial fibrillation among adults in the United States: REasons for Geographic and Racial Differences in Stroke (REGARDS) Study. Circ. Arrhythmia Electrophysiol..

[7] Olesen J.B., Lip G.Y., Kamper A.-L., Hommel K., Køber L., Lane D.A., Lindhardsen J., Gislason G.H., Torp-Pedersen C. (2012). Stroke and bleeding in atrial fibrillation with chronic kidney disease. N. Engl. J. Med..

[8] Hart R.G., Pearce L.A., Aguilar M.I. (2007). Meta-analysis: Antithrombotic therapy to prevent stroke in patients who have nonvalvular atrial fibrillation. Ann. Intern. Med..

[9] Connolly S.J., Pogue J., Hart R.G., A Pfeffer M., Hohnloser S.H., Chrolavicius S., Yusuf S., ACTIVE Writing Group of the ACTIVE Investigators (2006). Clopidogrel plus aspirin versus oral anticoagulation for atrial fibrillation in the Atrial fibrillation Clopidogrel Trial with Irbesartan for prevention of Vascular Events (ACTIVE W): A randomised controlled trial. Lancet.

[10] Shameem R., Ansell J. (2013). Disadvantages of VKA and requirements for novel anticoagulants. Best Pract. Res. Clin. Haematol..

[11] Connolly S.J., Eikelboom J., Joyner C., Diener H.-C., Hart R., Golitsyn S., Flaker G., Avezum A., Hohnloser S.H., Diaz R. (2011). Apixaban in patients with atrial fibrillation. N. Engl. J. Med..

[12] Connolly S.J., Ezekowitz M.D., Yusuf S., Eikelboom J., Oldgren J., Parekh A., Pogue J., Reilly P.A., Themeles E., Varrone J. (2009). Dabigatran versus warfarin in patients with atrial fibrillation. N. Engl. J. Med..

[13] Patel M.R., Mahaffey K.W., Garg J., Pan G., Singer D.E., Hacke W., Breithardt G., Halperin J.L., Hankey G.J., Piccini J.P. (2011). Rivaroxaban versus warfarin in nonvalvular atrial fibrillation. N. Engl. J. Med..

[14] Hindricks G., Potpara T., Dagres N., Arbelo E., Bax J.J., Blomström-Lundqvist C., Boriani G., Castella M., Dan G.-A., Dilaveris P.E. (2021). 2020 ESC Guidelines for the diagnosis and management of atrial fibrillation developed in collaboration with the European Association for Cardio-Thoracic Surgery (EACTS). Eur. Heart J..

[15] January C.T., Wann L.S., Alpert J.S., Calkins H., Cigarroa J.E., Cleveland Jr J.C., Yancy C.W. (2014). 2014 AHA/ACC/HRS guideline for the management of patients with atrial fibrillation: Executive summary: A report of the American College of Cardiology/American Heart Association Task Force on practice guidelines and the Heart Rhythm Society. Circulation.

[16] Freedman B., Potpara T.S., Lip G.Y. (2016). Stroke prevention in atrial fibrillation. Lancet.

[17] Chan K.E., Giugliano R.P., Patel M.R., Abramson S., Jardine M., Zhao S., Perkovic V., Maddux F.W., Piccini J.P. (2016). Nonvitamin K Anticoagulant Agents in Patients With Advanced Chronic Kidney Disease or on Dialysis With AF. J. Am. Coll. Cardiol..

[18] Landmesser U., Tondo C., Camm J., Diener H.C., Paul V., Schmidt B., Hildick-Smith D. (2018). Left atrial appendage occlusion with the AMPLATZER Amulet device: One-year follow-up from the prospective global Amulet observational registry. EuroIntervention J. EuroPCR Collab. Work. Group Interv. Cardiol. Eur. Soc. Cardiol..

[19] Baman J.R., Mansour M., Heist E.K., Huang D.T., Biton Y. (2018). Percutaneous left atrial appendage occlusion in the prevention of stroke in atrial fibrillation: A systematic review. Heart Fail. Rev..

[20] Ostermayer S.H., Reisman M., Kramer P.H., Matthews R.V., Gray W.A., Block P.C., Omran H., Bartorelli A.L., Della Bella P., Di Mario C. (2005). Percutaneous left atrial appendage transcatheter occlusion (PLAATO System) to prevent stroke in high-risk patients with non-rheumatic atrial fibrillation: Results from the international multi-center feasibility trials. J. Am. Coll. Cardiol..

[21] Reddy V.Y., Sievert H., Halperin J., Doshi S.K., Buchbinder M., Neuzil P., Holmes D. (2014). Percutaneous left atrial appendage closure vs warfarin for atrial fibrillation: A randomized clinical trial. JAMA.

[22] Reddy V.Y., Doshi S.K., Kar S., Gibson D.N., Price M.J., Huber K. (2017). 5-Year Outcomes after Left Atrial Appendage Closure: From the PREVAIL and PROTECT AF Trials. J. Am. Coll. Cardiol..

[23] Holmes D.R., Kar S., Price M.J., Whisenant B., Sievert H., Doshi S.K., Reddy V.Y. (2014). Prospective randomized evaluation of the Watchman Left Atrial Appendage Closure device in patients with atrial fibrillation versus long-term warfarin therapy: The PREVAIL trial. J. Am. Coll. Cardiol..

[24] Nielsen-Kudsk J.E., Korsholm K., Damgaard D., Valentin J.B., Diener H.C., Camm A.J., Johnsen S.P. (2021). Clinical Outcomes Associated with Left Atrial Appendage Occlusion versus Direct Oral Anticoagulation in Atrial Fibrillation. Cardiovasc. Interv..

[25] Whitlock R.P., Belley-Cote E.P., Paparella D., Healey J.S., Brady K., Sharma M., Reents W., Budera P., Baddour A.J., Fila P. (2021). Left Atrial Appendage Occlusion during Cardiac Surgery to Prevent Stroke. N. Engl. J. Med..

[26] Osmancik P., Herman D., Neuzil P., Hala P., Taborsky M., Kala P., Poloczek M., Stasek J., Haman L., Branny M. (2020). Left Atrial Appendage Closure Versus Direct Oral Anticoagulants in High-Risk Patients With Atrial Fibrillation. J. Am. Coll. Cardiol..

[27] Saw J., Holmes D.R., Cavalcante J.L., Freeman J.V., Goldsweig A.M., Kavinsky C.J., Moussa I.D., Munger T.M., Price M.J., Reisman M. (2023). SCAI/HRS Expert Consensus Statement on Transcatheter Left Atrial Appendage Closure. JACC: Cardiovasc. Interv..

[28] Glikson M., Wolff R., Hindricks G., Mandrola J., Camm A.J., Lip G.Y.H., Fauchier L., Betts T.R., Lewalter T., Saw J. (2020). EHRA/EAPCI expert consensus statement on catheter-based left atrial appendage occlusion—An update. Europace.

[29] Korsholm K., Valentin J.B., Damgaard D., Diener H.-C., Camm A.J., Landmesser U., Hildick-Smith D., Johnsen S.P., Nielsen-Kudsk J.E. (2022). Clinical outcomes of left atrial appendage occlusion versus direct oral anticoagulation in patients with atrial fibrillation and prior ischemic stroke: A propensity-score matched study. Int. J. Cardiol..

[30] Zeitler E.P., Kearing S., Coylewright M., Nair D., Hsu J.C., Darden D., O’malley A.J., Russo A.M., Al-Khatib S.M. (2023). Comparative Effectiveness of Left Atrial Appendage Occlusion Versus Oral Anticoagulation by Sex. Circulation.

[31] Lega J., Bertoletti L., Gremillet C., Boissier C., Mismetti P., Laporte S. (2014). Consistency of safety profile of new oral anticoagulants in patients with renal failure. J. Thromb. Haemost..

[32] Chan K.E., Lazarus J.M., Thadhani R., Hakim R.M. (2009). Anticoagulant and Antiplatelet Usage Associates with Mortality among Hemodialysis Patients. J. Am. Soc. Nephrol..

[33] Van Der Meersch H., De Bacquer D., De Vriese A.S. (2017). Vitamin K antagonists for stroke prevention in hemodialysis patients with atrial fibrillation: A systematic review and meta-analysis. Am. Heart J..

[34] Siontis K.C., Zhang X., Eckard A., Bhave N., Schaubel D.E., He K., Nallamothu B.K. (2018). Outcomes Associated with Apixaban use in Patients with End-Stage Kidney Disease and Atrial Fibrillation in the United States. Circulation.

[35] Lee S., Monz B.U., Clemens A., Brueckmann M., Lip G.Y.H. (2012). Representativeness of the dabigatran, apixaban and rivaroxaban clinical trial populations to real-world atrial fibrillation patients in the United Kingdom: A cross-sectional analysis using the General Practice Research Database. BMJ Open.

[36] Bertaglia E., Anselmino M., Zorzi A., Russo V., Toso E., Peruzza F., Rapacciuolo A., Migliore F., Gaita F., Cucchini U. (2017). NOACs and atrial fibrillation: Incidence and predictors of left atrial thrombus in the real world. Int. J. Cardiol..

[37] Wang K.-L., Lopes R.D., Patel M.R., Büller H.R., Tan D.S.-Y., Chiang C.-E., Giugliano R.P. (2018). Efficacy and safety of reduced-dose non-vitamin K antagonist oral anticoagulants in patients with atrial fibrillation: A meta-analysis of randomized controlled trials. Eur. Heart J..

[38] Sharma S.P., Park P., Lakkireddy D. (2018). Left Atrial Appendages Occlusion: Current Status and Prospective. Korean Circ. J..

