# Supplementary material for: Left Atrial Appendage Occlusion versus Novel Oral Anticoagulation for Stroke Prevention in Atrial Fibrillation—One-Year Survival
Source: J Clin Med. 2023 Oct 23;12(20):6693. doi: 10.3390/jcm12206693 (PMC10607551; doi:10.3390/jcm12206693)
Supplement: Supplementary file 1 [file jcm-12-06693-s001.zip › jcm-2572115-supplementary.pdf]

Left Atrial Appendage Occlusion versus Novel Oral Anticoagulation for Stroke Prevention in Atrial Fibrillation – One Year Survival

Tiosano et al.

Supplementary material

Contents

Table S1 – Baseline characteristics of study participants with CrCl <= 60 mL/min 2

Table S2 – The association between LAAO vs. NOACs and 1-year mortality – stratification by distinct groups. 3

Table S1 – Baseline characteristics of study participants with CrCl <= 60 mL/min

	LAAO *N=62*	NOACs *N=217*	P
**Age**	79.0±7.97	79.5±11.5	0.736
**Sex: Male**	31 (50.0%)	121 (55.8%)	0.510
**Hypertension**	50 (80.6%)	172 (79.3%)	0.953
**Diabetes Mellitus**	26 (41.9%)	81 (37.3%)	0.610
**Ischemic Heart Disease**	36 (58.1%)	108 (49.8%)	0.313
**Congestive Heart Failure**	25 (40.3%)	86 (39.6%)	1.000
**Ejection fraction (%)**	52.8±7.69	52.6±11.1	0.873
**Prior bleeding**	46 (74.2%)	168 (77.4%)	0.719
**Prior stroke or TIA**	31 (50.0%)	85 (39.2%)	0.168
**CrCl**	35.8±20.0	37.3±17.0	0.610
**CHA_2DS_2-VASc**	4.39±1.51	4.16±1.64	0.310
**CHA_2DS_2-VASc => 4**	47 (75.8%)	157 (72.4%)	0.705
**HAS-BLED**	4.13±1.18	4.38±1.27	0.153
**HAS-BLED => 4**	45 (72.6%)	185 (85.3%)	0.034
**Prior Aspirin treatment**	23 (37.1%)	74 (34.1%)	0.775

LAAO: Left Atrial Appendage Occlusion; NOACs: Novel Oral Anticoagulants; TIA: Transient Ischemic Attack; CrCl: Creatinine Clearance, mL/min

Table S2 – The association between LAAO vs. NOACs and 1-year mortality – stratification by distinct groups.

	LAAO	NOACs
**Under age 80**	6/174 (3.45%)	2/62 (3.23%)
**Age 80 plus**	26/168 (15.5%)	3/52 (5.77%)
**Males**	14/202 (6.93%)	4/70 (5.71%)
**Females**	18/140 (12.9%)	1/44 (2.27%)
**CrCl 60 plus**	3/125 (2.40%)	3/52 (5.77%)
**CrCl under 60**	29/217 (13.4%)	2/62 (3.23%)

LAAO: Left Atrial Appendage Occlusion; NOACs: Novel Oral Anticoagulants; CrCl: Creatinine Clearance
